# Ischemic Conditioning Ameliorated Hypertension and Vascular Remodeling of Spontaneously Hypertensive Rat via Inflammatory Regulation

**DOI:** 10.14336/AD.2020.0320

**Published:** 2021-02-01

**Authors:** Yu Gao, Changhong Ren, Xiaohua Li, Wantong Yu, Sijie Li, Haiyan Li, Yan Wang, Dong Li, Ming Ren, Xunming Ji

**Affiliations:** ^1^Department of Neurology, Xuanwu Hospital, Capital Medical University, Beijing, 10053, China.; ^2^Beijing Key Laboratory of Hypoxia Conditioning Translational Medicine, Xuanwu Hospital, Capital Medical, Beijing, 10053, China.; ^3^Beijing municipal geriatric medical research center, Beijing, 10053, China.; ^4^Peking University Care Health Management Center, Beijing, 100080, China.; ^5^Department of Neurosurgery Xuanwu Hospital, Capital Medical, Beijing 100053, China.

**Keywords:** hypertension, vascular remodeling, inflammation, limb remote ischemic preconditioning

## Abstract

Vascular remodeling is an initial step in the development of hypertension. Limb remote ischemic conditioning (LRIC) is a physiological treatment that induces endogenous protective effect during acute ischemic injury. However, the impact of long-term LRIC on hypertension, a chronic disease, is unknown. In this study, we aimed to investigate the LRIC effect on blood pressure and vascular remodeling in spontaneously hypertensive rat (SHR) model and patients with prehypertension and early-stage hypertension. LRIC of rats was performed once a day for 6-weeks. Blood pressure, vascular remodeling (cross-sectional area, extracellular deposition, and smooth muscle cell area), inflammation (inflammatory factors, and inflammatory cells) were compared among normotensive Wistar-Kyoto rats (WKY), WKY RIC group, SHR control group, and SHR RIC. Long-term LRCI treatment (twice a day for 4-weeks) was performed on patients with prehypertension or early-stage hypertension. Blood pressure and pulse wave velocity (PWV) were analyzed before and after LRIC treatment. LRIC treatment decreased blood pressure in SHR (n = 9-10). LRIC ameliorated vascular remodeling by decreasing cross-sectional area, suppressing deposition of the extracellular matrix, and hypertrophy of smooth muscle cell in conduit artery and small resistance artery (n = 7). LRIC decreased proinflammatory factors while increasing the anti-inflammatory factors in the circulation (n = 5). LRIC decreased circulating monocyte and natural killer T-cell levels (n = 5). Furthermore, LRIC treatment decreased blood pressure and improved vascular stiffness in patients (n = 20). In conclusion, long term LRIC could decrease blood pressure and ameliorate vascular remodeling via inflammation regulation. LRIC could be a preventive treatment for people with blood pressure elevation or prehypertension.

Hypertension is a leading risk factor for cardiovascular, cerebrovascular, and many other diseases [1], especially cardiovascular and cerebrovascular diseases, which are leading causes of global deaths, according to the World Health Organization [2]. Vascular remodeling and dysfunction are the main pathological processes preceding the occurrence of hypertension and its complications [3-5]. Vascular remodeling results from blood pressure elevation, and progressively becomes a crucial cause of hypertension [6]. However, during vascular remodeling no symptoms except occasional blood pressure elevation are observed. Neither attention nor treatment would be considered until the diagnosis of hypertension is established [7]. Thus, timely therapy is urgent for the prevention and treatment of early-stage vascular remodeling.

Vascular remodeling results initially from passive physiological adaptation to blood pressure changes, then progresses into an active pathological process caused by elevated blood pressure, aging, and several other factors [8, 9]. This pathological process involves a series of changes, including cell proliferation, migration, transformation, production of the extracellular matrix, and inflammation[5]. An earlier study showed the critical role of inflammation in the pathological changes involved in vascular remodeling [10]. Emerging evidence indicates that infiltrating proinflammatory cells are essential for the migration and infiltration of inflammatory factors [11]. Several inflammatory factors were shown to affect blood pressure and vascular function leading to vascular remodeling and dysfunction [12]. Thus, it is reasonable to hypothesize that by regulating inflammatory cells and their environment might aid in the treatment and prevention of vascular remodeling and hypertension-related vascular diseases.

Limb remote ischemic conditioning (LRIC) is a physiological treatment that protects against acute ischemic events and traumatic injury [13, 14]. Chronic remote ischemic conditioning simulates regular exercise and exerts its protective effect via humoral and immunological regulation [14, 15]. Some clinical cases reported that LRIC could decrease blood pressure [16]. However, studies on whether LRIC positively affects chronic vascular remodeling and blood pressure are scant. Considering all the available evidence, we hypothesize that LRIC would exert a protective effect on hypertension-related vascular remodeling, thus delaying vascular stiffness and aging caused by structural remodeling.

In this study, we aimed to address whether LRIC exhibits antihypertensive effect and if LRIC prevents or delays vascular remodeling in a chronic hypertensive pathological condition and the possible underlying mechanism of LRIC protective effect.

## MATERIALS AND METHODS

### Animals

Spontaneously hypertensive rats (SHR) were used as a genetic model of hypertension, which develop hypertension at a very young age. Before the established phase, the blood pressure of SHR increases, and vascular remodeling occurs rapidly from age 4-10 weeks [17]. In this study, we chose the 4-week-old SHR, which has similar blood pressure compared to the Wistar-Kyoto rats (WKY), normotensive controls.

The Animal Care and Use Committee of Xuanwu Hospital, Capital Medical University, China approved all animal experiments which were conducted following the National Institutes of Health guidelines. Forty rats used in this study were purchased from the Vital River Laboratories, Beijing, China, and maintained on a 12-h light/dark cycle with unlimited access to food and water.

We assigned randomly 20 WKY to WKY control (WKY-CON) and WKY + RIC groups, and 20 SHR to SHR control (SHR-CON) and SHR + RIC groups, respectively.

### Limb Remote Ischemic Conditioning

**Figure 1.**
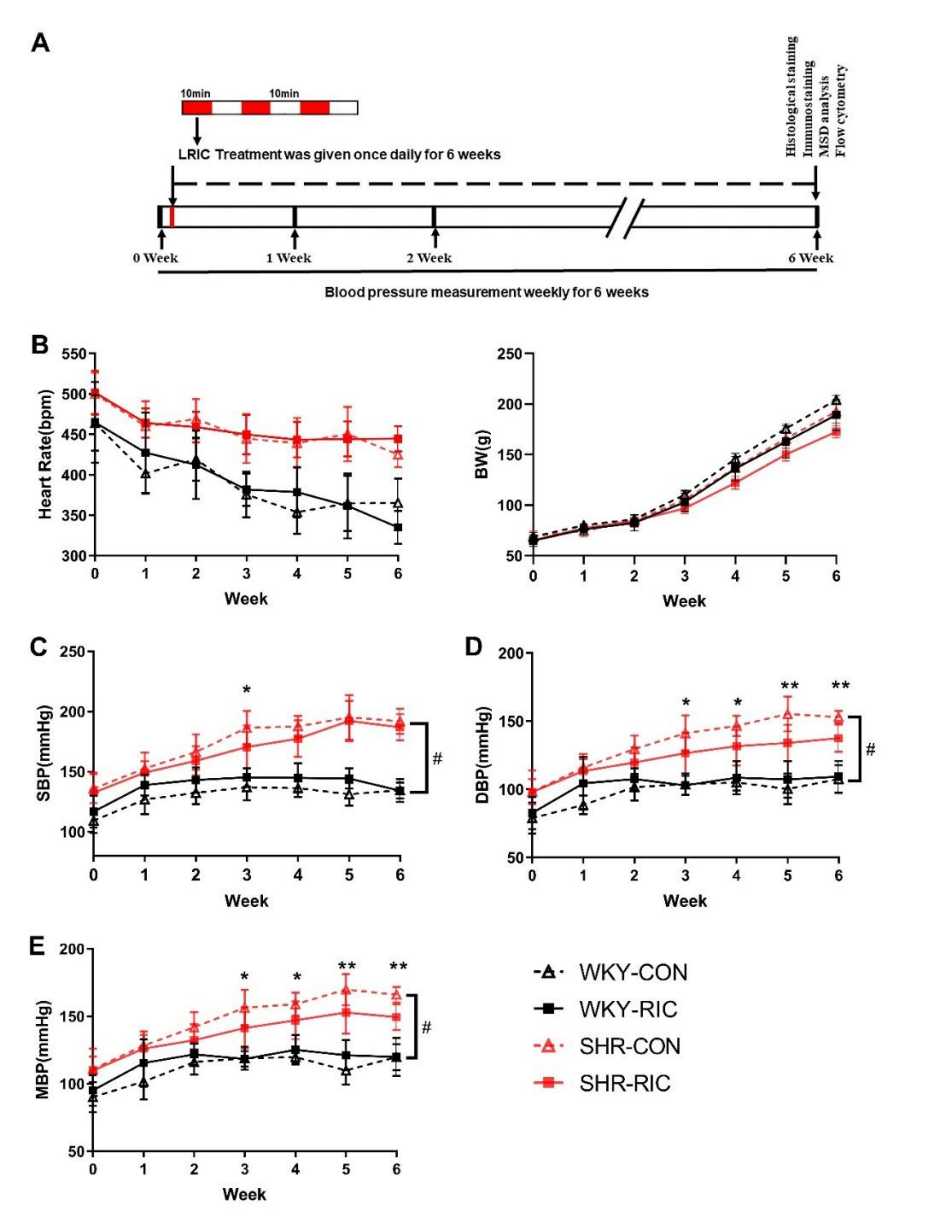
Effect of LRIC on blood pressure in SHR and WKY. (A) Representative sketches of this experiment. (B) Heart rate and the bodyweight of each group. Systolic blood pressure (SBP) (C), diastolic blood pressure (DBP) (D), and mean blood pressure (MBP) (E) of each group over 6-weeks. Data are expressed as mean ± SD, * P<0.05, * * P<0.01, * * * P<0.001, SHR control vs. SHR RIC. # P<0.01, WKY control vs. SHR control, n = 9-10 each group.

LRIC was conducted as previously described[18]. Rats were anesthetized with an intraperitoneal injection of sodium pentobarbital (30 mg/kg), and three cycles of LRIC conducted daily by tightening and releasing of a tourniquet around the upper thigh. Each cycle comprised of 10 min of tightening (ischemia) and 10 min of release (reperfusion). The treatment was administered every day for 6-weeks (Fig. 1A).

### Blood Pressure Measurements

The blood pressure was measured weekly, 12 hours after LRIC, with a tail-cuff system (SoftronBP-2010A; Softron, Tokyo, Japan) following the manufacturer’s guidelines.

### Histological analysis

The thoracic aorta, second-order mesenteric artery, and brain were harvested and fixed in 4% paraformaldehyde, dehydrated, and embedded in paraffin. Paraffin sections of 5 µm thickness were deparaffinized and washed, stained with hematoxylin and eosin (H&E), Sirius-red, and Verhoeff's elastic stain (VEG) [19]. For H&E staining, tissues were incubated with hematoxylin solution for 8 min, rinsed in running water, differentiated in 1% acid alcohol for 30 sec, rinsed in water, and counterstained in eosin solution for 1 min. Sirius-red staining was carried out by incubating tissues with Sirius red for 1 h, washed, and nuclei stained with Mayer’s solution followed by washing. For VEG staining, tissue was immersed in Verhoeff’s solution for 1 h, differentiated with 2% ferric chloride for 1 min, washed in water, then treated with 5% sodium thiosulfate for 1 min, washed, and counterstained with Van Gieson’s solution for 3-5 min. Then all slices were dehydrated in alcohol, cleared in xylene, and mounted using xylene based mounting medium. Images were viewed and captured using a digital slide scanner (3DHISTECH, UK) and Nikon Ti Eclipse Epi-fl Illuminator (Nikon, Japan). The images were quantified by Image J software. The quantification of hypertrophic remodeling includes the medial area and vessel wall thickness. The collagen and elastic fiber content were calculated by summing up all positive tissue areas from all measurements of the section [19].

### Immunohistochemistry

Tissue slices were incubated overnight at 4°C with anti-SMA antibody (1:100, Abcam, US). After washing with PBS, the sections were incubated for 1 h at 21 °C with the secondary antibodies. Slides were mounted with an antifade reagent containing DAPI (Molecular Probes, USA). Fluorescence signals were detected with the Panoramic digital slide scanner (3DHISTECH, UK), and quantified using Image J software. The α-SMA expression values were calculated from the total positive area and mean fluorescence intensity.

### Flow Cytometry

Immunocytes in the blood were analyzed by flow cytometry. Single-cell suspensions were treated with Fc block, washed, and stained with CD3, CD4, CD8, CD45, CD161a, CD43, CD172a, and their homologous isotype-matched negative controls (BD, Franklin Lakes, NJ). Live cells were gated, and a set number of events were acquired using a Fortessa flow cytometer (BD) and analyzed.

### Measurement of Inflammatory Factors

Blood from the inferior vena cava was collected into EDTA coated vacutainer tubes, followed by centrifugation at 1000g for 15 min at 21 °C. Plasma was obtained and stored at -80°C [20]. An ELISA assay for plasma inflammatory factors was conducted with the Meso Scale Discovery (MSD, US) rat inflammation kit following the manufacturer’s instructions.

**Table 1:** Baseline characteristics of recruited patients.

Characteristics	Value, n = 20
Age	57 ± 8.4
Male	9 (45)
Height, Kg	68.9 ± 8.8
Weight, cm	166.1 ± 8.3
BMI (Kg/m^2^)	24.8 ± 2.4
Systolic blood pressure, mmHg	137.2 ± 9.5
Diastolic blood pressure, mmHg	82.9 ± 6.9
Mean blood pressure, mmHg	100.8 ± 6.1
Heart rate, bpm	73.5 ± 8.1
Cholesterol	
Total, mmol/L	5.3 ± 0.8
LDL, mmol/L	2.9 ± 0.6
HDL, mmol/L	1.4 ± 0.2
Triglycerides, mmol/L	1.9 ± 0.9
Creatinine, µmol/L	5.8 ± 0.6
Fasting blood glucose, mmol/L	4.6 ± 1.4
White blood cell count (10^9^)	5.5 ± 1.1

BMI, body mass index; LDL, low-density lipoprotein; HDL, high-density lipoprotein. All the parameters are mean ± SD except Male, n (%).

### Patient recruitment and treatment

The Institutional Ethics Committee of Xuanwu Hospital approved this investigation. In this study, 20 pre-hypertensive or early-stage hypertensive subjects not receiving any pharmacological anti-hypertensive treatment were included. The study inclusion criteria were as follows: (1) systolic blood pressure (SBP) between 125-160 mmHg or diastolic blood pressure (DBP) between 80-100 mmHg; (2) age ≥18; (3) essential hypertension; (4) patient not receiving any anti-hypertensive medication. The exclusion criteria were: (1) patients with severe uncontrolled diabetes; (2) contraindication for remote ischemic preconditioning; (3) life expectancy less than one year; (4) patients with atrial fibrillation or other kinds of arrhythmia; (5) unwillingness for follow-up or poor compliance. (Registration No. NCT04254432, https://register.clinicaltrials.gov)

The baseline characteristics of patients are shown in Table 1. LRCI treatment was administered as described previously, and all patients habituated to the treatment over three days [21]. Bilateral upper limb RIC was performed using an electric auto-control device (patent number ZL200820123637.X, China), which consisted of five alternating cycles of inflation and deflation for 5 minutes, twice daily for 4-weeks, and the inflation pressure was 200 mmHg.

Baseline blood pressure and pulse wave velocity (PWV) were measured following the previously described guidelines [1]. Patients were prepared accordingly, and the blood pressure measured by electronic blood pressure monitor (Omron, Japan) and PWV measured by the non-invasive Vascular Screening Device (Omron, Japan). After completion of the LRIC treatment, blood pressure and PWV were measured within 3 days.

### Statistical analysis

Data calculated using SPSS version 19.0 (SPSS, Chicago, IL, USA) software were expressed as mean ± standard deviation (SD). For animal experiments, differences between groups were analyzed using one-way analysis of variance (ANOVA), and posthoc multiple comparisons were performed using Fisher’s LSD where appropriate. Two-way repeated-measures ANOVA was used for analysis of a comparison of blood pressure across time points. For the human trials, differences between groups were analyzed using two-sided paired Student’s *t*-test. All analyses were performed with significance set at P<0.05.

## RESULTS

### LRIC treatment decreased SHR blood pressure with no effect on heart rate and body weight

Blood pressure in SHR increased rapidly from the age of 4-10 weeks compared to the WKY group. The mean blood pressure of the two groups was 119.8 ± 9.6 and 166.0 ± 5.7 mmHg, respectively (Fig. 1E). LRIC treatment decreased SHR blood pressure to certain extent without affecting body weight and heart rate (Fig. 1B). The mean blood pressure in the SHR-CON and SHR RIC groups was 166.0 ± 5.7 and 149.5 ± 9.6 mmHg, respectively, after a 6-week treatment. LRIC resulted in decreased blood pressure of greater than 10 mmHg compared to SHR-CON group. The effect of LRIC on blood pressure appeared in the third week of treatment and continued after that (Fig. 1E). However, LRIC could not decrease SHR blood pressure to levels comparable to WKY.

**Figure 2.**
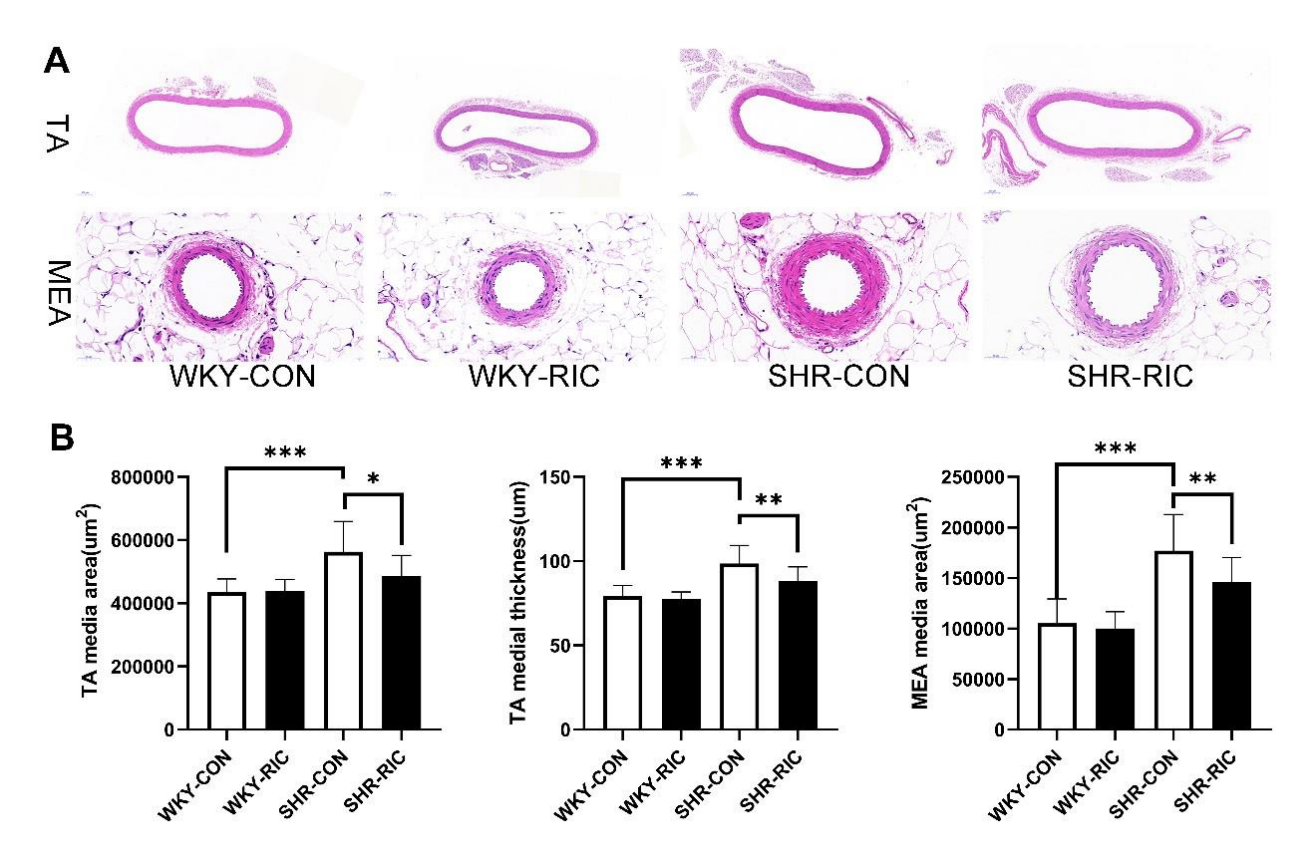
LRIC treatment ameliorated hypertrophic vascular remodeling of conducting artery and small resistance artery. (A) H&E staining of the thoracic aorta (TA) and second-order mesenteric artery (MEA). (B) Tunica media area and the wall thickness of TA and MEA of each group. Data are expressed as mean ± SD, * P<0.05, * * P<0.01, * * * P<0.001, WKY-CON vs. SHR-CON, SHR-CON vs. SHR-RIC. N = 7 each group.

### LRIC treatment ameliorated hypertrophic vascular remodeling of conducting artery and small resistance artery (second-order mesenteric artery)

Hypertrophy is recognized as the primary cause during the transition from the physiological to pathological vascular remodeling in hypertension [5]. Hypertrophic remodeling is visible primarily in the increased media cross-sectional area and decreased media/lumen ratio. We investigated whether LRIC has a positive effect on vascular remodeling by performing Hematoxylin and Eosin (H&E) staining on the thoracic aorta and small resistance artery (second-order mesenteric artery). The area of media and wall thickness was evaluated after a 6-week treatment (Fig. 2A). The media area of the thoracic aorta and small resistance artery of SHR increased significantly compared to WKY (Fig. 2B). LRIC treatment decreased media thickness and media area of the thoracic aorta and second-order mesenteric artery by 10 and 18%, respectively, compared to SHR-CON (Fig. 2B) The LRIC showed a higher beneficial effect on small resistance artery than that on conducting artery (Fig. 2B).

### LRIC treatment ameliorated hypertrophic vascular remodeling of brain resistance artery (middle cerebral artery, MCA, and basilar artery, BA)

**Figure 3.**
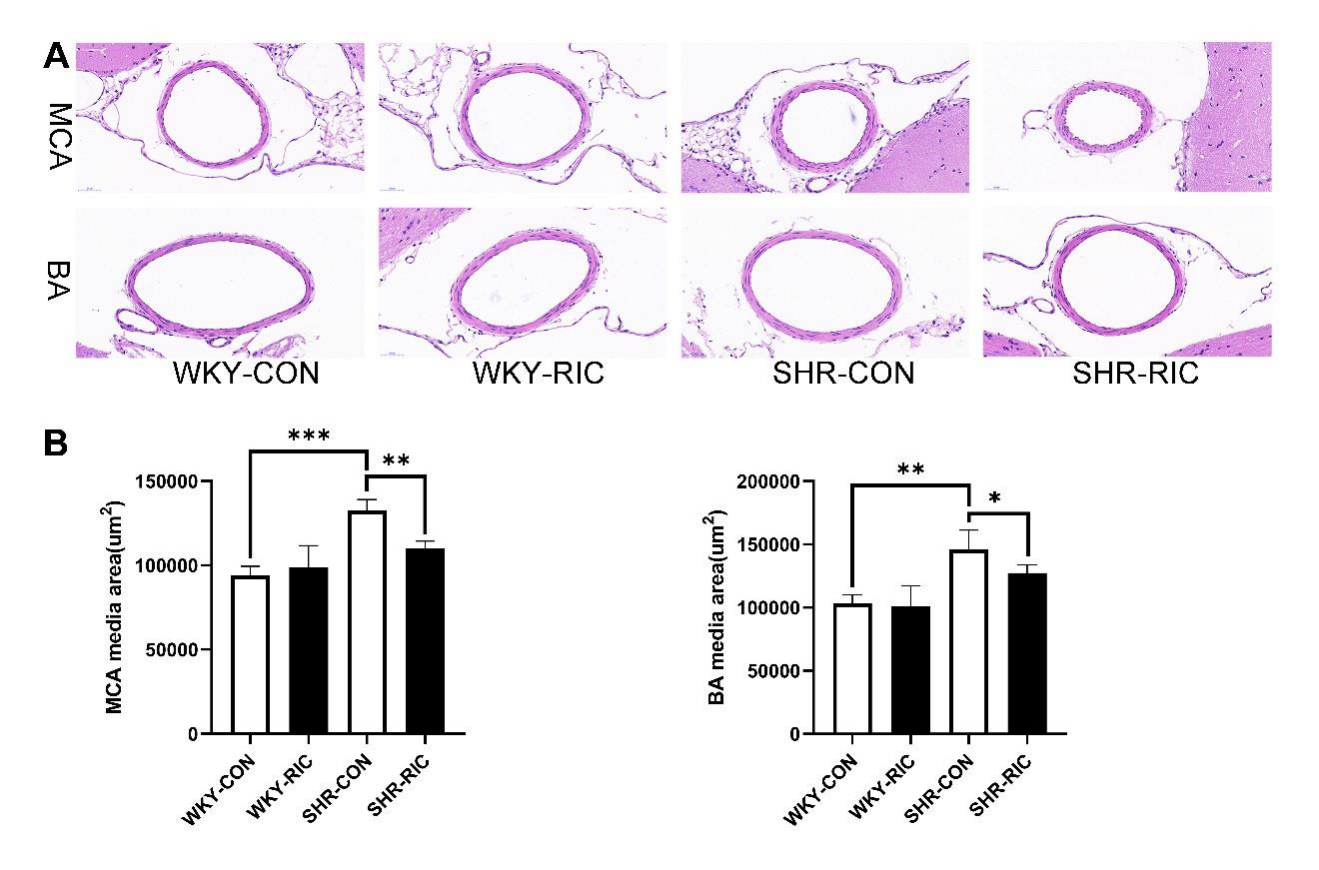
LRIC treatment ameliorated hypertrophic vascular remodeling of middle cerebral artery and basilar artery. (A) H&E staining of the middle cerebral artery (MCA) and basilar artery (BA). (B) Media area of MCA and BA of each group. Data are expressed as mean ± SD, * P<0.05, * * P<0.01, * * * P<0.001, WKY-CON vs. SHR-CON, SHR-CON vs. SHR-RIC. N = 5 each group.

Hypertrophic remodeling causes increased peripheral resistance and decreased blood supply [12]. The resistance of muscular arteries appears to be higher in the cerebral circulation than in other vascular systems [23]. The cerebral resistance artery contributes considerably to total cerebral vascular resistance in normal and hypertensive conditions, which further decreases cerebral blood supply and brain dysfunction [22, 23]. We assessed whether LRIC exhibited a similar effect on the cerebral resistance artery compared to systemic by performing H&E staining on the main cerebral resistance artery, middle cerebral artery (MCA), and basilar artery (BA) and compared the media area of each group (Fig. 3A). Cerebral resistance arteries of SHR showed significant hypertrophic remodeling compared to WKY (Fig. 3B). LRIC could ameliorate the hypertrophic vascular remodeling of MCA and BA, decreased the media area by 17 and 13%, respectively (Fig. 3B). The effect was similar to that on the mesenteric artery.

### LRIC suppress extracellular matrix production of the conducting artery and small resistance artery

**Figure 4.**
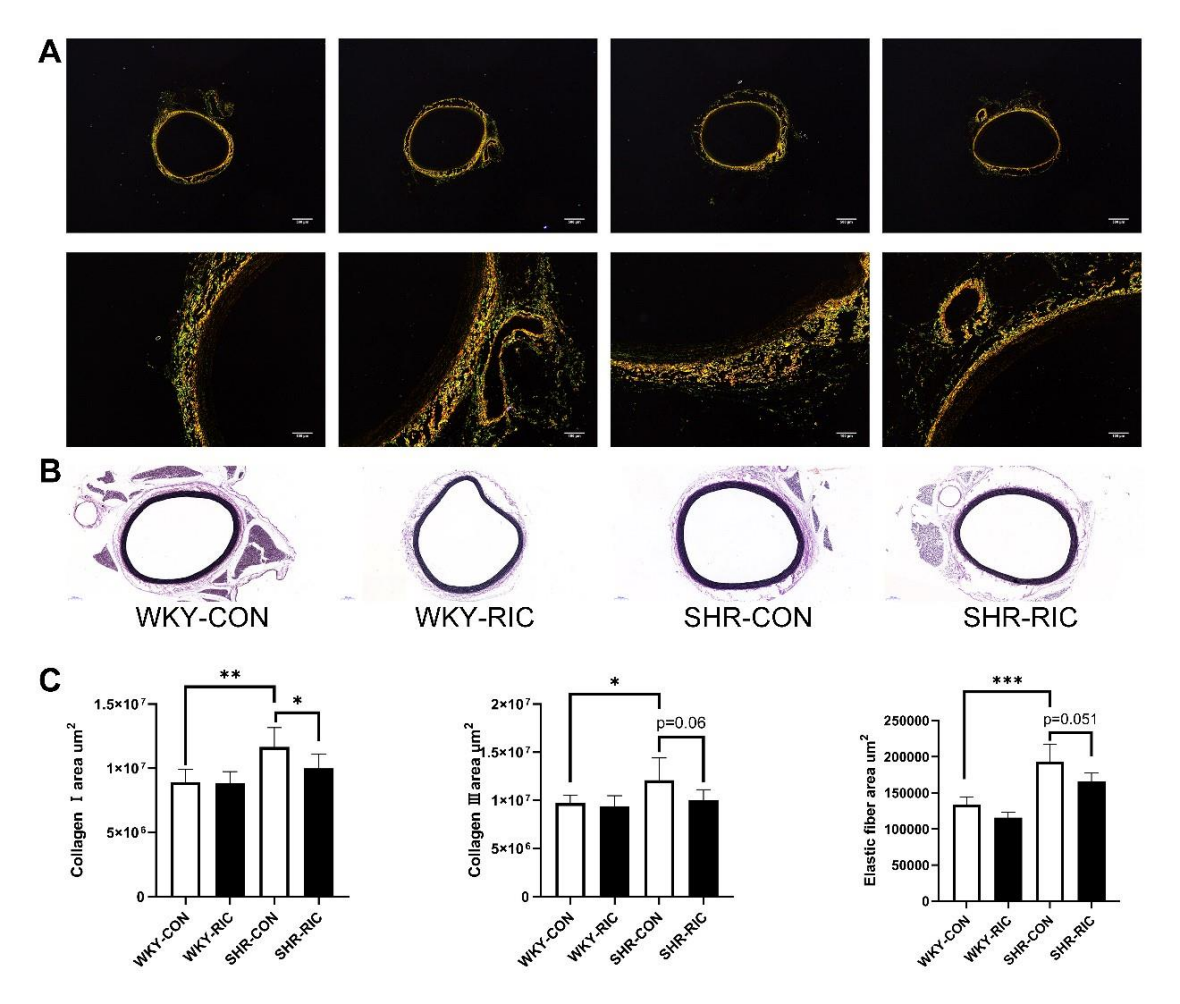
LRIC suppressed the production of the extracellular matrix of the conducting artery. Sirius-red (A) and VEG staining (B) of TA. (C) Collagen I, Collagen III, and elastic fiber area of TA. Data are expressed as mean ± SD, * P<0.05, * * P<0.01, * * * P<0.001, WKY-CON vs. SHR-CON, SHR-CON vs. SHR-RIC. N = 7 each group.

**Figure 5.**
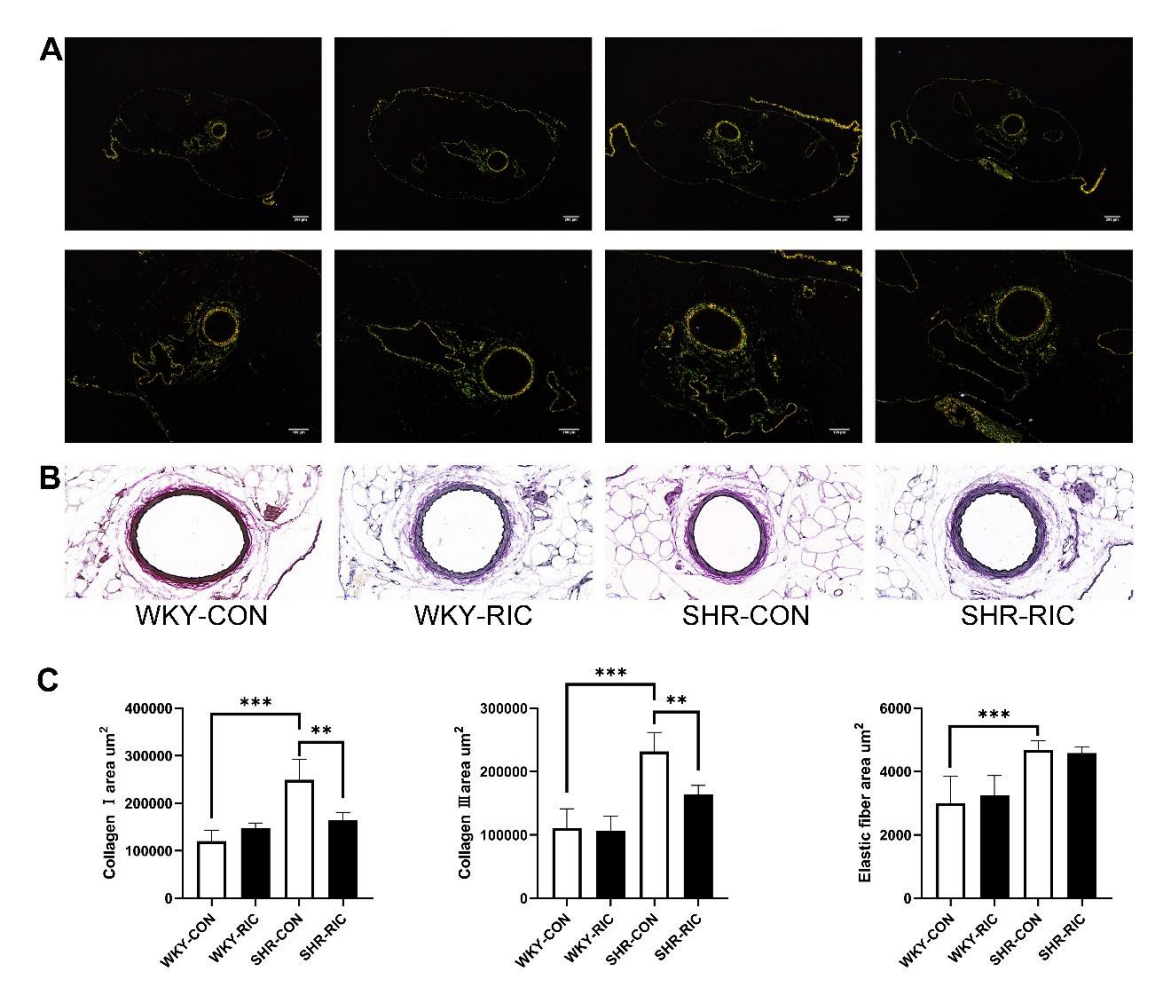
LRIC suppressed the production of the extracellular matrix of the small resistance artery. Sirius-red (A) and VEG staining (B) of MEA. (C) Collagen I, Collagen III, and elastic fiber area of MEA. Data are expressed as mean ± SD, * P<0.05, * * P<0.01, * * * P<0.001, WKY-CON vs. SHR-CON, SHR-CON vs. SHR-RIC. N = 7 each group.

Vascular hypertrophy involves extracellular matrix production, smooth muscle cell hyperplasia, and hypertrophy [24]. We investigated their effect on the cross-sectional area by examining levels of different extracellular matrices, collagen Ⅰ, collagen Ⅲ, and elastic fiber from each group (Fig. 4A and B; Fig. 5A and B). The results showed markedly increased deposition of collagen Ⅰ, collagen Ⅲ, and elastic fiber in the SHR-CON group compared to the WKY-CON (Fig. 4C; Fig. 5C). LRIC decreased both collagen Ⅰ, and collagen Ⅲ deposition in TA and MEA (Fig. 4C; Fig. 5C). Collagen levels decreased by 30 and 15% in MEA and TA, respectively, and LRIC showed a negligible 3% decrease in the elastic fiber of MEA (Fig. 5C). In contrast, LRIC decreased by nearly 15% of elastic fiber deposition in TA (Fig. 4C). Altogether, LRIC suppresses the production of collagen in the small resistance artery considerably with no significant effect on the elastic fiber. LRIC decreased 15% of all extracellular component production in the conduit artery.

### LRIC treatment suppressed smooth muscle cell hypertrophy of small resistance artery

**Figure 6.**
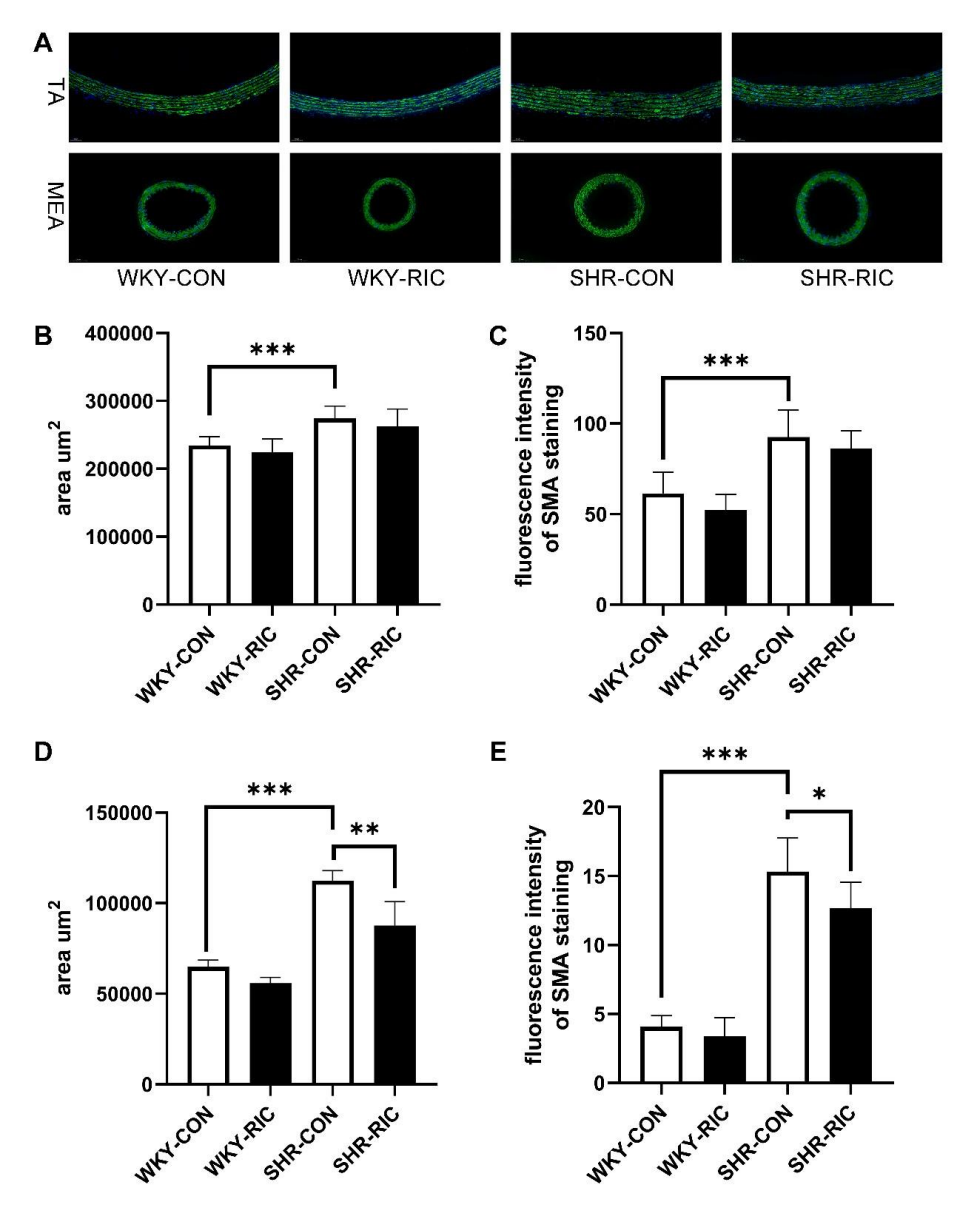
LRIC suppressed smooth muscle cell hypertrophy of small resistance artery. (A) Immunostaining of smooth muscle actin of the TA and MEA. SMC area (B) and fluorescence intensity (C) in TA of each group. SMC area (D) and fluorescence intensity (E) in MEA of each group. Data are expressed as mean ± SD, * P<0.05, * * P<0.01, * * * P<0.001, WKY-CON vs. SHR-CON, SHR-CON vs. SHR-RIC. N = 5 each group.

Smooth muscle cell (SMC) hypertrophy and hyperplasia were reported in early-stage hypertension and play a significant role in pathological remodeling [5]. Hypertrophy is considered a critical cellular response process in early hypertension [25]. We investigated whether LRIC would affect SMC by immunostaining of α-SMA in the vascular smooth muscle cell (Fig. 6A). Results showed that the area and expression of α-SMA increased significantly in the SHR-CON compared to WKY-CON (Fig. 6B, C, D, E). The area and fluorescence intensity of SMC decreased by 20% and 5% in MEA and TA, respectively. α-SMA expression decreased by 17% and 7% in MEA and TA, respectively. These results indicated that LRIC could ameliorate SMC hypertrophy in the small resistance artery with no effect on the conducting artery.

### LRIC treatment regulated circulating pro-inflammatory and anti-inflammatory factors

**Figure 7.**
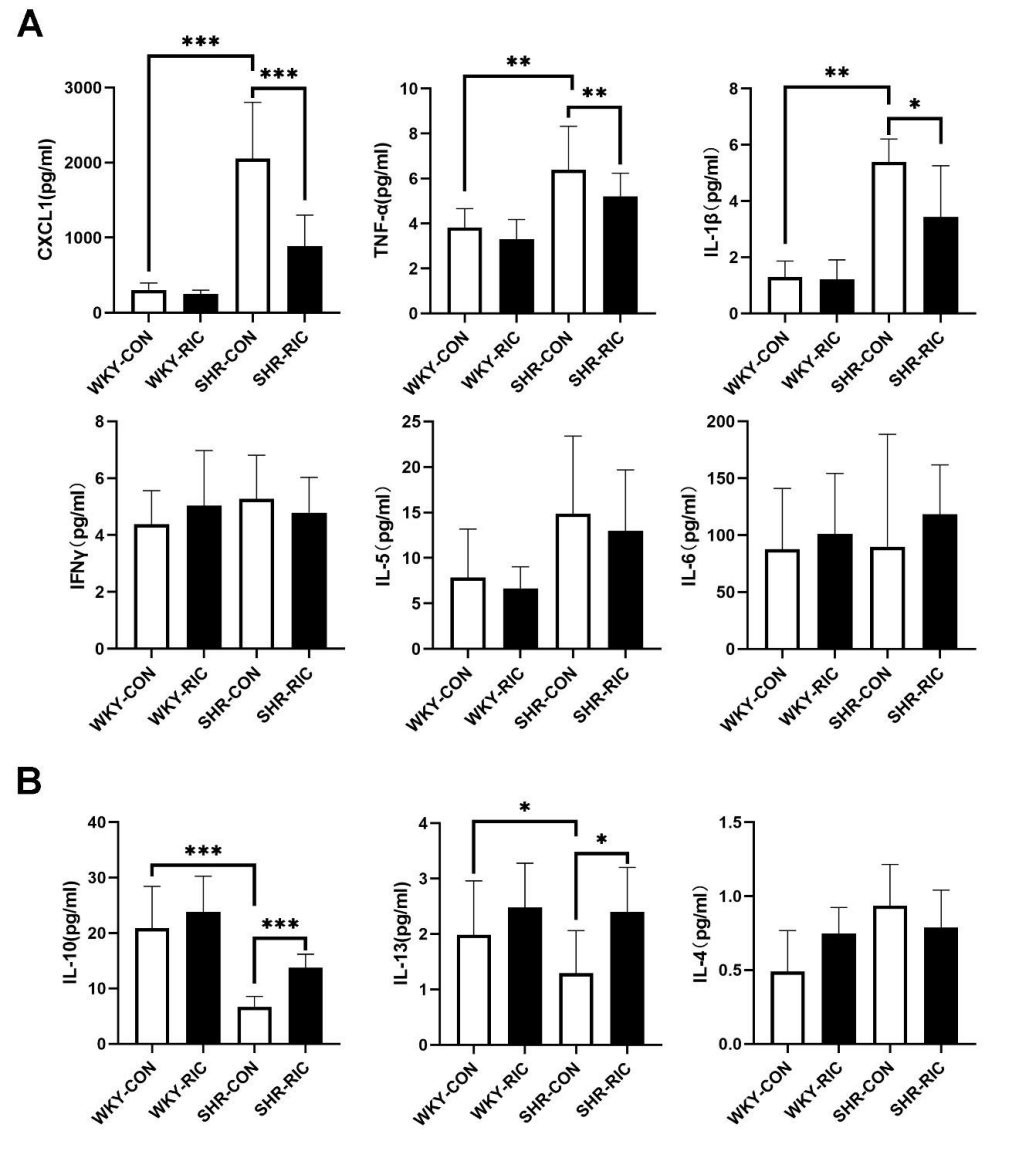
LRIC treatment regulated circulating pro-inflammatory and anti-inflammatory factors. (A) pro-inflammatory cytokines CXCL1, TNFα, IL-1β, IFNγ,IL-5, and IL-6 levels in plasma. (B) anti-inflammatory cytokines IL-10, IL-13, and IL-4 levels in plasma. Data are expressed as mean ± SD, * P<0.05, * * P<0.01, * * * P<0.001, WKY-CON vs. SHR-CON, SHR-CON vs. SHR-RIC. N = 5 each group.

Dysregulation of circulating inflammatory factors was shown to play a central role in initiating blood pressure elevation and early-stage vascular remodeling [12]. The effect of LRIC on circulating inflammatory factors was investigated by determining the levels of hypertension-related pro- and anti-inflammatory factors using MSD. The results showed significantly increased pro-inflammatory factors CXCL1, TNFα,and IL-1β in SHR (Fig. 7A), compared to WKY, while the anti-inflammatory factors IL-10 and IL-13 decreased markedly (Fig. 7B). The levels of other inflammatory cytokines, IFNγ, IL-5, IL-6, and IL-4, were not significantly different between SHR vs. WKY (Fig. 7A). LRIC decreased pro-inflammatory factors CXCL1, TNFα, and IL-1β (Fig. 7A) and increased anti-inflammatory factors IL-10 and IL-13 in SHR (Fig. 7B). LRIC treatment did not affect the levels of IFNγ, IL-4, IL-5, and IL-6 (Fig. 7 A and B).

### LRIC treatment regulated inflammatory immunocytes in circulation

**Figure 8.**
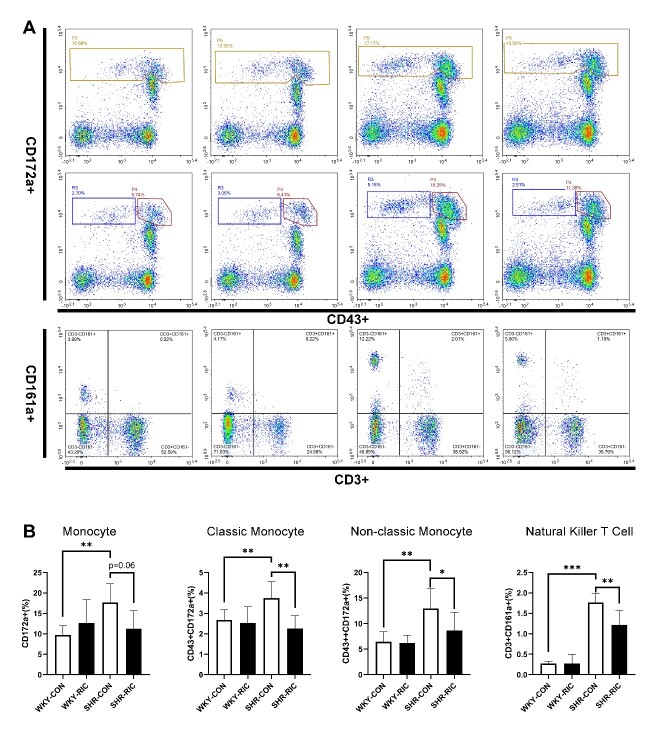
LRIC treatment regulated inflammatory immunocytes in circulation. (A) Flow cytometry analysis of CD172a+, CD43+ 172a+, CD43++ 172a+, and CD3+CD161a+ cells in the circulation. (B) level of monocyte, classic monocyte, non-classic monocyte, and natural killer T cells in circulation of each group. Data are expressed as mean ± SD, * P<0.05, * * P<0.01, * * * P<0.001, WKY-CON vs. SHR-CON, SHR-CON vs. SHR-RIC. N = 5 each group.

Hypertrophic remodeling is initiated by vascular infiltration of inflammatory cells, especially macrophages [11]. Inflammatory factors secreted by immunocytes are critical components involved in recruitment and infiltration during the pathogenesis of vascular remodeling in hypertension [26]. Thus, we analyzed the immunocyte levels in each group to investigate whether LRIC affects immunity (Fig. 8A). We found an increased number of all types of monocytes in SHR compared to WKY (Fig. 8B). In addition, we identified significantly increased natural killer T cells (NKT) in SHR (Fig. 8B). LRIC decreased levels of monocytes and natural killer T cells significantly (Fig. 8B).

### Long term LRIC decreased blood pressure and ameliorated conducting artery PWV

**Figure 9.**
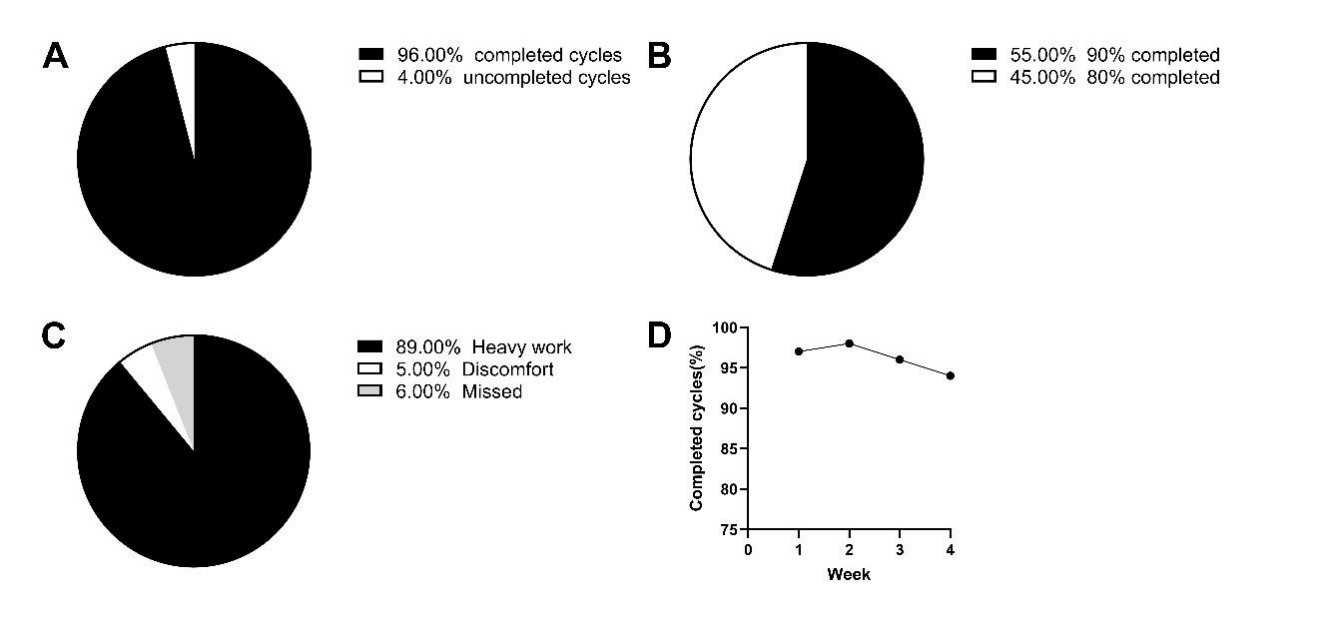
Hypertensive patient tolerance of LRIC. (A) The percentage of completed and uncompleted cycles. (B) The percentage of 90% and 80% completed patients. (C) The reasons for uncompleted cycles. (D) The percentage of completed cycles in each week.

**Figure 10.**
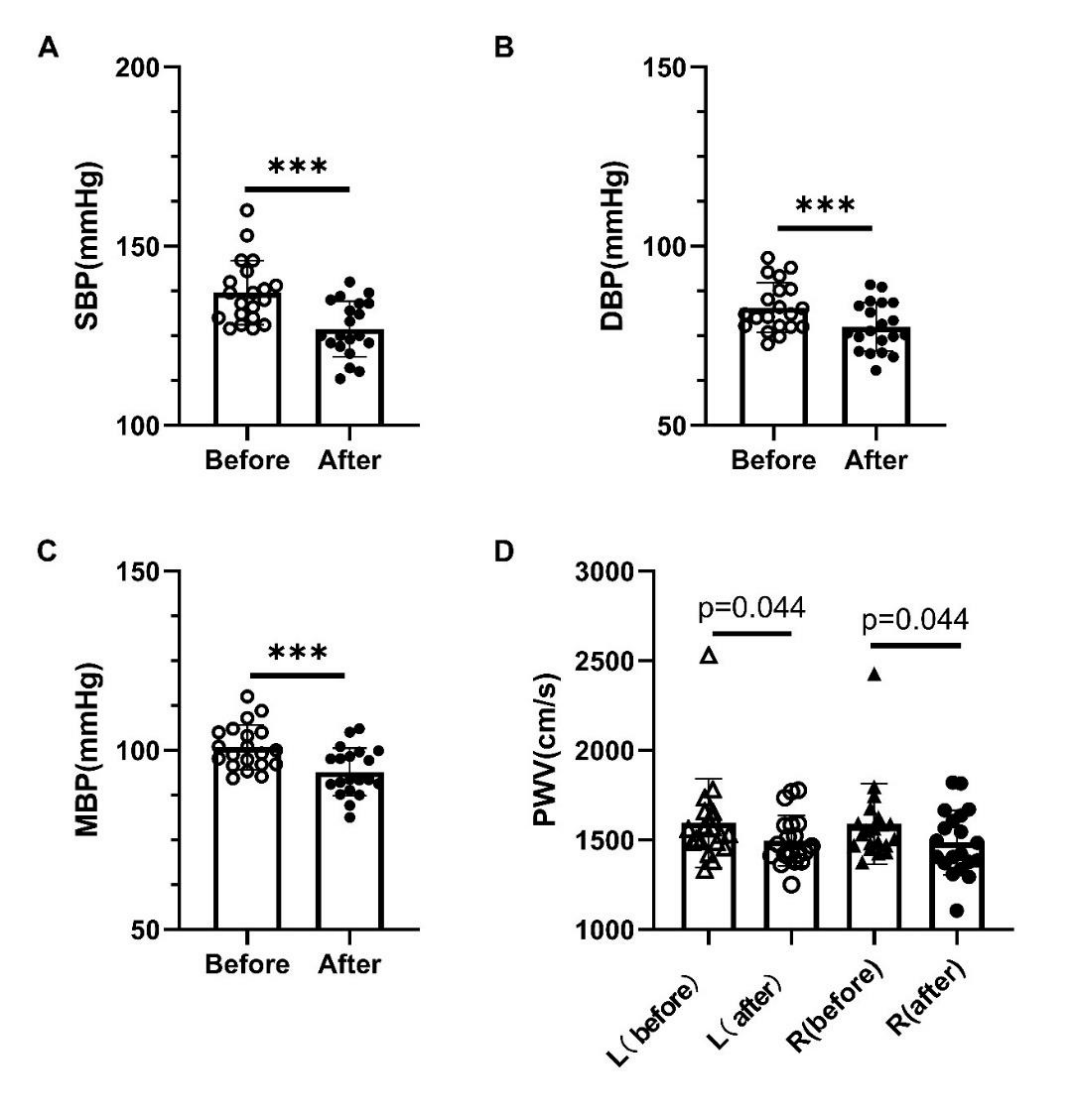
Long term LRIC decreased blood pressure and ameliorated conducting artery PWV. (A) Systolic blood pressure before and after LRIC treatment. (B) Diastolic blood pressure before and after LRIC treatment. (C) mean blood pressure before and after LRIC treatment. (D) PWV of left and right sides before and after LRIC treatment. * P<0.05, * * P<0.01, ***P<0.001, before vs. after.

We investigated whether LRIC exhibits similar effects in humans by conducting a pilot study on patients with pre- and early-stage hypertension. Twenty patients completed the 4-week ischemic conditioning treatment. The results showed that a 4-week LRIC treatment was well tolerated: 5476 out of 5600 cycles were completed in total. Eleven and nine subjects completed over 90 and 80% cycles, respectively. The percentage of completed cycles for each week was 98%, 97%, 96%, and 94%, respectively. The primary reason for uncompleted cycles was intense workouts, while other reasons are, discomfort due to cuff pressure, or lack of adherence to schedule (Fig. 9). The systolic blood pressure (SBP), diastolic blood pressure (DBP), and mean blood pressure (MBP) decreased by 10.2, 5.4, and 6.78 mmHg, respectively, after a 4-week LRIC treatment compared to their baseline blood pressure. PWV decreased 101 cm/s on average (Fig. 10). The results showed that long-term LRIC could decrease blood pressure and ameliorate the large artery stiffness in patients with pre- or early-stage hypertension.

## DISCUSSION

In this study, the results indicated that SHR develops hypertension coupled with vascular remodeling as early as 4-week old, as reported previously [17]. Long-term LRIC could decrease blood pressure to a certain extent; however, it could not reverse to normal levels. Histological staining results demonstrated that LRIC treatment ameliorated hypertrophic vascular remodeling of the conducting artery, resistance artery, and cerebral resistance artery, which contribute to considerable resistance to cerebral circulation than any other organ. LRIC could suppress hypertrophic vascular remodeling in two ways. One is to suppress the production of extracellular matrix; the other is to suppress SMA hypertrophy and hyperplasia. LRIC reduced inflammatory factors and increased anti-inflammatory factors in circulation. Consistent with the inflammatory analysis, LRIC reduced monocyte count in the blood, a source of inflammatory factors. In addition, LRIC decreased blood pressure and PWV, indicators of arterial remodeling [27]. Altogether, this study suggested that LRIC could lower blood pressure by regulating the circulating inflammatory cells and their markers to ameliorate hypertension-induced vascular remodeling. It is noteworthy that LRIC could decrease blood pressure and reduce arterial stiffness, thus offering a promising approach to the prevention of hypertension and vascular remodeling.

Vascular remodeling underlies the pathological basis for all types of vascular diseases, and pathological remodeling helps establish stable hypertension [28]. Hypertensive vascular remodeling includes eutrophic remodeling and hypertrophic remodeling. Hypertrophic remodeling plays a central role in the development of hypertension and hypertensive end-organ injury [12]. For the conduit artery, hypertrophic remodeling is described as an outward increase of cross-sectional area and stiffness, similar to aging [29]. In the present study, we showed that LRIC could ameliorate the large artery remodeling primarily by suppressing deposition of the extracellular matrix. Consistent with the results, we found that long term LRIC could improve vascular stiffness in patients with pre- and early-stage hypertension. These results confirmed further that LRIC could protect the conducting artery from the pathological remodeling induced by hypertension or aging. The hypertrophic changes in the resistance artery refer mainly to an inward increase of cross-sectional area and decreased lumen, important components in end-organ damage, and most prevalent in early-stage hypertension [29, 30]. LRIC was shown to ameliorate the small resistance artery remodeling by suppressing the production of collagen and hypertrophy of smooth muscle cell. In addition, LRIC had a significant effect on the small resistance artery and cerebral muscular artery than that on the conduction artery. It is suggested that LRIC might be useful in the prevention and amelioration of diseases resulting from small artery remodeling like the cerebral small vessel disease [21].

Accumulating evidence demonstrates that increased circulating pro-inflammatory factors initiate vascular remodeling pathogenesis at an early-stage, typically accompanied by a decrease in anti-inflammatory factors [31]. Inflammation dysregulation could elevate blood pressure and trigger vascular remodeling. Suppressing pro-inflammatory factors or their receptors could decrease blood pressure and prevent vascular remodeling [32]. Hypertension is one such stimulus that initiates vascular remodeling mediated by inflammatory cell migration and infiltration [12].

CXCL1 is a chemokine that promotes migration and infiltration of neutrophils and monocytes to vascular wall[31]. The blockage of CXCR2, the receptor of CXCL1, could suppress hypertension and end-organ damage in SHR and other hypertensive animal models [32, 33]. Our results showed that LRIC could significantly decrease plasma CXCL1 levels, suggesting that LRIC ameliorated vascular remodeling by decreasing CXCL1 production. TNFα has been reported to lead to the development of hypertension in various hypertensive models[34]. Anti-TNFα therapy reduced vascular inflammation and reduced arterial stiffness in patients with autoimmune diseases[35]. IL-1β levels were higher in hypertension and mediated vascular inflammation [36]. Consistent with previous results, our results demonstrated an increase in TNFα and IL-1β levels in SHR and LRIC reduced plasma TNFα and IL-1β levels, indicating that LRIC exerts its effect by suppressing pro-inflammatory marker expression.

IL-10 is an anti-inflammatory cytokine exhibiting a protective effect on vascular dysfunction in hypertension [37]. A significant increase in IL-10 after kidney ischemic preconditioning was reported earlier[38]. IL-13 and IL-4 induced polarization of macrophage cells to an M2 phenotype, which expresses anti-inflammatory markers [39]. Previous research has found an imbalance in M1 and M2 population in SHR with an increased M2 macrophage population resulting in the normalization of blood pressure [40]. In this study, we found that LRIC increased circulating IL-10 and IL-13, which might participate in improving vascular function. All the findings in this study suggested that LRIC ameliorated vascular remodeling by suppressing the production of multiple pro-inflammatory factors while inducing the production of anti-inflammatory factors in the circulation. Elevated inflammatory status exists in various chronic diseases, including aging [41], and LRIC opens a promising avenue for the prevention of aging and chronic diseases.

Circulating monocyte forming macrophages entering a specific organ or tissue plays a critical role in vascular inflammation and remodeling [42]. It is found that decreased number of lysozyme M-positive monocytes in circulation ablated the hypertensive stimuli induced vascular infiltration [11]. In addition, monocytes/ macrophages secrete pro-inflammatory factors and express receptors to interact with chemokines, such as CXCL1, TFNα, and IL-1β [43]. Consistent with the inflammatory factor analysis, the monocyte counts in the circulation decreased after LRIC treatment. We also observed decreased classic monocyte counts compared to non-classic monocytes. Classic monocytes express pro-inflammatory clusters and produce pro-inflammatory factors, TNFα and IL-1β[44]. From our results, it is suggested that LRIC could suppress the monocyte counts, especially the pro-inflammatory subset involved in hypertension and vascular remodeling. It is noteworthy that LRIC therapy could decrease the NKT cell level significantly. The relationship between NKT cells and hypertension has not been elucidated yet [45]. A previous study reported the association of NKT cells with cardiovascular events, while others demonstrated attenuation of vascular remodeling by a specialized NKT cell subset [46]. We showed markedly higher NKT cell counts in SHR compared to WKY, which decreased dramatically after LRIC treatment, thus shedding light on the novel mechanism of hypertension development.

Successful blood pressure management is critical to prevent cardiovascular and cerebrovascular disease. Some studies showed that sustained elevated blood pressure exceeding 125 mmHg might represent a threshold for vascular remodeling, potential hypertension, and increased risk of future cardiovascular disease and stroke [47]. All the evidence highlights the importance of early prevention of blood pressure elevation. Our study results showed that LRIC could reduce the onset of blood pressure during pre- and early-stages in humans, suggesting that LRIC offers other means of hypertension and vascular dysfunction prevention for clinical translation.

However, some limitations exist in the human study. First, this is a single-arm study, and the date was not compared with the control group. It cannot exclude placebo effects, which may affect blood pressure. Instead, the data before and after ischemic conditioning were self-compared. In addition, the method used to evaluate blood pressure is not precise, and the effect on blood pressure should be explored in a longer time frame. Thus, the results from the human study are implied and are being investigated further in a randomized, double-blind placebo control study (Registration No. NCT03566654, https://register.clinicaltrials.gov).

In conclusion, long-term LRIC treatment could decrease blood pressure and attenuate vascular remodeling in SHR. LRIC exerted its protective effect by restoring the imbalance in pro- and anti-inflammatory markers. These findings describe the potential use of LRIC for the prevention of hypertension.
